# Development of immortalized human hepatocyte-like hybrid cells by fusion of multi-lineage progenitor cells with primary hepatocytes

**DOI:** 10.1371/journal.pone.0234002

**Published:** 2020-06-04

**Authors:** Daniel P. Collins, Joel H. Hapke, Rajagopal N. Aravalli, Clifford J. Steer

**Affiliations:** 1 CMDG, LLC, Saint Paul, Minnesota, United States of America; 2 Department of Electrical and Computer Engineering, College of Science and Engineering, University of Minnesota, Minneapolis, Minnesota, United States of America; 3 Department of Medicine, University of Minnesota Medical School, Minneapolis, Minnesota, United States of America; 4 Department of Genetics, Cell Biology and Development, University of Minnesota Medical School, Minneapolis, Minnesota, United States of America; Università degli Studi della Campania, ITALY

## Abstract

Human primary hepatocytes (PHs) are critical to studying liver functions, drug metabolism and toxicity. PHs isolated from livers that are unacceptable for transplantation have limited expansion and culture viability *in vitro*, in addition to rapidly deteriorating enzymatic functions. The unsuitability of immortalized hepato-carcinoma cell lines for this function has prompted studies to develop hepatocyte-like cells from alternative sources like ESC, iPS, and other stem cell types using differentiation protocols. This study describes a novel technique to produce expandable and functional hepatocyte-like cells from the fusion of an immortalized human umbilical cord blood derived cell line (E12 MLPC) to normal human primary hepatocytes. Multi-lineage progenitor cells (MLPC) comprise a small subset of mesenchymal-like cells isolated from human umbilical cord blood. MLPC are distinguishable from other mesenchymal-like cells by their extended expansion capacity (up to 80 cell doublings before senescence) and the ability to be differentiated into cells representative of endo-, meso- and ectodermal origins. Transfection of MLPC with the gene for telomerase reverse transcriptase (TERT) resulted in clonal cell lines that were capable of differentiation to different cellular outcomes while maintaining their functional immortality. A methodology for the development of immortalized hepatocyte-like hybrid cells by the *in vitro* fusion of human MLPC with normal human primary hepatocytes is reported. The resultant hybrid cells exhibited homology with hepatocytes by morphology, immunohistochemistry, urea and albumin production and gene expression. A medium that allows stable long-term expansion of hepatocyte-like fusion cells is described.

## Introduction

Human primary hepatocytes (PH) play a crucial role in the study of liver diseases, the development of new therapies, and are the standard for toxicological studies of drug metabolism. Currently the source of primary hepatocytes for these studies are obtained from donor liver deemed unsuitable for transplantation. Limitations of the use of PH include (i) brief *in vitro* viability with diminishing enzymatic activity over time; and (ii) large variability between donor hepatocytes with regards to plate-ability, enzymatic activity, and toxicological reactivity. Additionally, PH have limited proliferative capacity, thus significantly reducing the availability of specific donor cells [1,2]. A stable cell line with the functionality of hepatocytes and the proliferative capacity to be greatly expanded *in vitro*, in combination with the potential for large-scale applications could be a useful tool for *in vitro* hepatocyte studies. In this study we report a novel methodology to create a long-lived cell line with the functional characteristics of PH by the *in vitro* fusion of an immortalized cord blood-derived stem cell with a primary human hepatocyte.

Multi-lineage stem cells (MLPC) are a primitive subset of mesenchymal-like stem cells isolated from human umbilical cord blood [3–8]. MLPC distinguish themselves from other mesenchymal-like cells by their ability to (i) be extensively expanded in culture (up to 80 population doublings); (ii) develop non-transformed clonal cell lines; and (iii) be differentiated to endo- and ectodermal lineages in addition to the mesodermal lineages attributed to other mesenchymal stem cell types.

Establishment of clonal cell lines from polyclonal MSC-like cells isolated from umbilical cord blood suggested that only 5–10% of MSC-like cells could be cloned and grown to significant quantities for study. The cells that were cloned and expanded demonstrated the ability to differentiate to non-mesodermal lineages while the non-clonable cells were restricted to mesodermal lineages only. Those cells that exhibited the qualities of expansion and differentiation were named MLPC (multi-lineage progenitor cells) [9–11]. After transfection of non-cloned MSC-like cord blood cells with the gene for hTERT and subsequent cloning, it was observed again that only 5–10% of cells were capable of differentiation to non-mesodermal cell types. They, however, were shown to be functionally immortalized and have been grown for over 12 years, while retaining their ability to be differentiated to endo-, meso- and ectodermal outcomes. The E12 clonal cell line that demonstrated the most differential capacity, was used throughout this study.

Using a methodology initially developed for to make monoclonal antibodies [12], immortalized E12 MLPCs were fused to normal human primary hepatocytes. This resulted in the establishment of a unique hybrid cell line that was expandable and capable of continuous culture while exhibiting the phenotype and biological activity of well-differentiated and mature human hepatocytes. This methodology could provide a pathway for the development of organ-, disease- or person-specific cells and organoids for drug or therapy development.

## Materials and methods

### Isolation of MLPC

Umbilical cord blood was collected as part of a study to develop PrepaCyte-CB, an FDA-allowed product to de-bulk cord blood for cryo-banking and transplantation for hematopoietic reconstruction after myeloablation. IRB approval of the studies was conducted by the University of Minnesota and the Saint Louis Cord Blood Bank. The cord blood samples were collected by the American Red Cross Cord Blood Program in Saint Paul, Minnesota and Ridgeview Medical Center (Waconia, MN). Donations were collected with donor consent for research use only with no identifiers available for the donors. Collection of human umbilical cord blood was IRB approved by Quorum Review Protocol #800, March 3, 2005. MLPC were a commercially available product market by BioE from 2006–2010.

Briefly, post-partum human cord blood was mixed at a one-to-one ratio with PrepaCyte-MSC (CMDG, LLC, Saint Paul, MN) for 30 minutes at room temperature and then allowed to sediment for 30 minutes in the same container. The sediment consisted of erythrocytes, monocytes, and granulocytes. The supernatant containing lymphocytes, hematopoietic stem cells and mesenchymal-like cells was removed and sedimented at 400 x g for 7 minutes. Cells were plated in MSCGM medium (Lonza, Walkerville, MD) at a concentration of 1.33 x 10^6^/cm^2^ and allowed to culture for 24 hours. After 24 hours the non-adherent cells were removed by washing and the medium was replaced with fresh MSCGM 3 times per week. Cells were harvested when cultures were 80–90% composed of cells with a fibroblastic morphology, and used to establish the clonal cell lines.

### Development of clonal cell lines

Generation of clonal populations of cells was achieved by serial dilution. Plastic-adherent cord blood-derived cells with a fibroblastic appearance were grown to ~ 90% purity. These cells were detached by trypsin or Tryp-LE and diluted to a concentration of 30 cells/20 ml of MSCGM. Cells were seeded in a 96-well tissue culture plate (200 μl per well) and were left to adhere overnight. Those wells with a single cell were expanded to 60–80% confluence, harvested and further expanded. Upon expansion, cultures were examined for a variety of surface markers to distinguish between MSC (bright CD90, negative for CD106 and CD31), MLPC (dull bimodal CD90, positive CD106, negative for CD31) and endothelial precursor cells (positive for CD90, CD106 and CD31). Establishment of clonal lines demonstrated the difference between MLPC and other mesenchymal-like cells found in cord blood. Non-MLPC mesenchymal-like cells senesced by 20 population doublings and were not capable of differentiation beyond mesodermal outcomes, while MLPC clones were capable of up to 80 population doublings and endo-, meso-, and ectodermal outcomes.

### hTERT vector production

pRRLsin.hCMV hTERT lentiviral expression plasmid was the kind gift of Dr. Noriyuki Kasahara, Department of Medicine, UCLA, Los Angeles, CA. The telomerase vector was produced by three plasmid transient transductions using 10μg of the self-inactivating (sin) hTERT lentiviral expression plasmid, 10μg of the gag/pol plasmid, pCMV delta 8.2, and 2μg of the envelope plasmid, pCMV VSVG, in a calcium phosphate transduction protocol according to the manufacturer’s directions (Clontech). HEK 293T cells at 60–70% confluence in a 10cm dish were given 10ml fresh medium (DMEM, 10% FBS without antibiotics) 3–4 hours prior to transduction. After incubation of transduced cells overnight at 37°C, 5%CO_2_, the medium was replaced with 6 ml fresh medium and incubated for an additional 24 hours. The supernatant was then collected and passed through a 0.45μm filter and stored at –80°C until used for transduction.

### Transduction of normal MLPC

Mixed MLPC cultures (passage 12) were seeded at a density of 5x10^4^ cells/well in a 6-well tissue culture dish in complete MSCGM (without antibiotics) 24-hours prior to transduction. The vector supernatant was diluted 1:10 with DMEM 10% FBS containing 8 μg/ml polybrene and 1 ml was added to each well from which the growth medium had been removed. After 4 hours at 37°C, 5% CO_2_, the diluted vector was removed and replaced with MSCGM. To estimate transduction efficiency, MLPC were also transduced in parallel with pRRL sinhCMV GFP vector supernatants, which had been serially diluted. GFP expression was analyzed 60 hours after transduction using FACS analysis. The titer of the GFP vector supernatant was 5x10^4^. Polyclonal TERT-transduced MLPC were the kind gift of Dr. Eve Kelland, Department of Neurology, Keck School of Medicine, University of Southern California, Los Angeles, CA.

### Establishment of MLPC-TERT cell lines

As with the non-transfected MLPC, clonal MLPC-TERT cell lines were developed by limited dilution cloning. Wells with only one detectable cell were propagated in larger culture vessels to achieve cell numbers sufficient for analysis. Of the ten stable cell lines that were developed, only one clone exhibited the combined characteristics of immortality and differentiation outside of mesodermal outcomes, E12-TERT (E12). This cell line has been in cultured for over 12 years and was used in this study.

### Primary hepatocytes

Cryo-preserved primary human hepatocytes were obtained from Zenotech (Kansas City, KS). Cells were thawed with OptiThaw medium and enumerated with OptiCount medium in a standard hemacytometer. Cells were diluted to a final concentration of 10^6^ cells/ml of OptiPlate medium. Cells were plated in collagen-coated 6 well plates (BD Biosciences) at 1 ml per well. After 4 hours of plating, the medium was changed to OptiCulture medium for the duration of culture. Medium was exchanged every 24 hours.

### Fusion of TERT-MLPC with primary human hepatocytes

TERT-MLPC (2–5 x 10^6^) are mixed with an equal number of primary human hepatocytes and pelleted at 300 x g for 5 minutes. Cells are washed twice with 10 ml RPMI + 0.01% EDTA. Cells are again pelleted and the supernatant removed. One ml of 50% polyethylene glycol in RMPI + 0.01% EDTA was added to the cells and the cells are gently resuspended. After one minute, 1 ml of RPMI+0.01% EDTA was added, and then after another minute 4 ml of RPMI + 0.01% EDTA was added both without mixing. After an additional 2 minutes 4 ml of RPMI + 20% fetal bovine serum (FBS) was added. Cells were pelleted by centrifugation at 50 x g for 10 minutes, diluted with 30 ml of RPMI + 20% FBS and resuspended. Fifteen ml of cell suspension were added to 75 cm^2^ collagen-coated tissue culture flasks. Cells adhered to the culture flask overnight and were cultured for 1 week in RPMI + 20% FBS with culture medium exchanged every other day. After 7 days, cells were examined by confocal microscopy for co-expression of TERT and albumin by immunohistochemistry, as described below. Unfused primary hepatocytes were non-viable after 7 days and did not contribute to resultant cell lines. If 100% of the cells were double positive for TERT and albumin, no further selection was performed. If less than 100% of the cells were double positive for TERT and albumin, cells were passaged by limiting dilution and re-tested for TERT and albumin until 100% of the cells were double positive. Of the 5 fusions performed, 2 fusions required no further selection, 3 fusions required only 2 passages before 100% expression of TERT and albumin. These cells were expanded by the method described in the following section and were used in the functional studies.

### Expansion of fusion cells

After stabilization of the fusion cells at 1 week of culture in RPMI + 20% FBS, cells isolated from the culture vessel were incubated with Tryp-LE (Life Technologies, Grand Island, NY), and then pelleted at 300 x g for 5 minutes. They were then resuspended at a concentration of 2 x 10^4^ cells/ml in Williams Medium E supplemented with 2% fatty acid-free BSA, 1% ITS solution, 5mM hydrocortisone 21-hemisuccinate, FGF basic (20 ng/ml), FGF-4 (20 ng/ml), HFG (40 ng/ml), SCF (40 ng/ml), Oncostatin M (20 ng/ml), BMP-4 (20 ng/ml), EGF (40 ng/ml) and IL-1 beta (20 ng/ml). Cells were grown to confluence prior to harvesting for cryopreservation or further expansion. Those used in the study had undergone a minimum of 4 cycles of expansion, cryopreservation, and re-expansion with 5 PD per expansion cycle.

### Confocal immunofluorescent analysis

Cells were harvested from their culture vessels by dissociation with Tryp-LE (Life Technologies, Grand Island, NY), counted and resuspended in the same expansion medium at a density of 10^5^ cells/ml. Two hundred μl of cell suspension was added to each well of a collagen-coated 16-well glass chamber slide (Nalge Nunc International, Rochester, NY). Cells were cultured overnight to facilitate adherence to the slide. After attachment, cells were fixed for 1 hour in 1% formalin, and permeabilized by PermaCyte Medium (CMDG, St. Paul, MN). Cells were incubated with ~ 100 ng of antibodies specific for alkaline phosphatase, alpha-fetoprotein, albumin, c-reactive protein, hepatocyte growth factor receptor, nestin, SOX-17, asialoglycoprotein receptor 1, hepatocyte nuclear factor-4, GATA-4 and alpha-1-antitrypsin (all from R&D Systems, Minneapolis, MN) coagulation factor VII, coagulation factor IX (from Invitrogen, Rockford, IL), P450 CYP 1A2, P450 CYP 3A4, glucuronosyltransferase isoforms UGT1A1, UGT2B7 (Abcam, Cambridge, MA), and TERT (Novus, Littleton, CO) for 40 minutes. Cells were then washed with PermaCyte to remove unbound antibody and stained with secondary antibodies specific for mouse or rabbit antibody labeled with Alexa 594 dye (Life Technologies, (Eugene, OR). Cells were counterstained with DAPI dye to visualize the nucleus of each cell. Positivity of staining was confirmed by comparison to cells stained with antibody isotype controls (CMDG, St. Paul, MN). Cells were then analyzed using the Olympus Fluoview 1000 confocal microscope.

### FISH analysis

Expression of the TERT gene in TERT-transfected cells was analyzed by fluorescent *in situ* hybridization using a QuantiGene ViewRNA probe set (Affimetrix, Santa Clara, CA). Briefly, MLPC-TERT cells were plated into 4 well chamber slides at a concentration of 1 x 10^5^ cells per well and allowed to adhere overnight. Using reagents included in the kit, cells were fixed with 1% Formalin for 1 hour, permeabilized with detergent for 15 minutes, treated with protease solution for 10 minutes to prepare for hybridization. Hybridization probe was added to sample and incubated for 3 hours at 40°C. Probe was removed by washing with PBS and the pre-amplifier mix was added and incubated for 30 minutes at 40°C. Pre-amplifier mix was removed by washing with PBS. Amplifier mix was added and incubated for 30 minutes at 40°C, after which amplifier mix was removed by washing with PBS. Label probe (Cy3 labeled) was added to cells and incubated for 30 minutes at 40°C. Excess label probe was removed by washing with PBS. Cells were counterstained with DAPI. Cells were analyzed by confocal microscopy on the Olympus Fluoview 1000 using the CY3 and DAPI filters.

### RT-PCR analysis

To test for expression of hepatocyte-specific genes in MLPC/hepatocyte fusion cells, total RNA was extracted from cells using the UltraPure™ Phenol:Chloroform:Isoamyl Alcohol reagent (Invitrogen) and Platinum® Quantitative RT-PCR ThermoScript ™ One-Step System (Invitrogen) was used to carry out quantitative RT-PCR reactions according to manufacturer’s recommendations. Total RNA isolated from E12 MLPC was used as the negative control, and that isolated from primary hepatocytes was used as a positive control. Primers are described for alpha-fetoprotein (AFP), α-1-antitrypsin (AAT), transthyretin (TTR), cytochrome P450 1A2 (CYP 1A2), cytochrome P450 3A4 (CYP 3A4), cytochrome P450 2C9 (CYP 2C9), hepatocyte nuclear factor 1α (HNF1A), hepatocyte growth factor (HGF), albumin (ALB), and housekeeping gene glyceraldehyde-3-phosphate dehydrogenase (GAPDH) [13–18]. The PCR primer sequences are listed on S1 Table. Conditions for PCR reactions were initial denaturation at 94°C for 3 min followed by 30 cycles of denaturation at 94°C for 1 min, annealing for 1 min at 56°C, and elongation for 1 min at 72°C. PCR products were then resolved using a 1% agarose gel, and visualized under UV light.

### Karyotypic analysis of fusion cells

Actively growing fusion cells were treated with colecemid overnight to arrest cells in metaphase. After, cells were harvested by standard cytogenetic protocol. They were treated with 0.75 M KCl hypotonic solution and fixed with 3:1 methanol:acetic acid. Cells were transferred to glass slides and stained with Wright’s/Geimsa. Twenty G-banded metaphase cells were analyzed and 2 were karyotyped. Cells were analyzed using an Olympus BX61 microscope. The imaging and karyotyping of metaphase chromosomes was performed using Applied Spectral Imaging Software.

### Urea production

Urea synthesis in MLPC/hepatocyte fusion cells was analyzed with a colorimetric assay (Biovision, Milpitas, CA). Cells were cultured for 3 days in the medium specific for each cell type. After 3 days, cells were dissociated from the culture plate using the Tryp-LE reagent, counted with hemocytometer and centrifuged at 400 x g for 5 minutes to pellet them. Supernatant was discarded and cells were re-suspended in 1 ml of WIF water (Millipore/Sigma). Cells were then exposed to three repeated freeze-thaw cycles of freezing at -80°C and thawing at room temperature to rupture cell membranes and release contents into the fluid phase. Cells were then centrifuged at 1000 x g for 10 minutes to pellet cell debris. Supernatant from the cell lysate was collected and analyzed by the urea assay, according to the manufacturer’s instructions (Biovision). Results were standardized to 1x10^6^ cells/ml of supernatant to enable comparison between cell culture samples with different cell numbers. Resultant samples were analyzed on the Emax microplate reader (Molecular Devices. San Jose, CA) and processed with SoftMax PRO 4.8 Analysis Software. E12 MLPC and PHs were used as negative and positive controls, respectively. At least four separate determinations were carried on different days.

### Albumin production

Albumin production was analyzed with an enzymatic ELISA assay (Abcam, Cambridge, MA). Cells were cultured for 3 days in the medium specific for each cell type, after which they were dissociated from the culture plate using the Tryp-LE reagent, counted with hemocytometer and pelleted at 400 x g for 5 minutes. Supernatant was discarded and cells were re-suspended in 1 ml of WIF water (Millipore/Sigma). Cells were then exposed to three repeated freeze-thaw cycles of freezing at -80°C and thawing at room temperature to rupture and release the intracellular contents into the fluid phase. Cells were then centrifuged at 1000 x g for 10 minutes to pellet the cell debris. Supernatant from the cell lysate was collected and analyzed by the albumin ELISA, according to the manufacturer’s instructions. Results were standardized to 1x10^6^ cells/ml of supernatant to enable comparison between cell culture samples with different cell numbers. Resultant samples were analyzed on the Emax microplate reader (Molecular Devices. San Jose, CA) and processed with SoftMax PRO 4.8 Analysis Software. E12 MLPC and PHs were used as negative and positive controls, respectively. A total of four separate determinations were carried on different days.

## Results

### Cellular morphology

**Fig 1:**
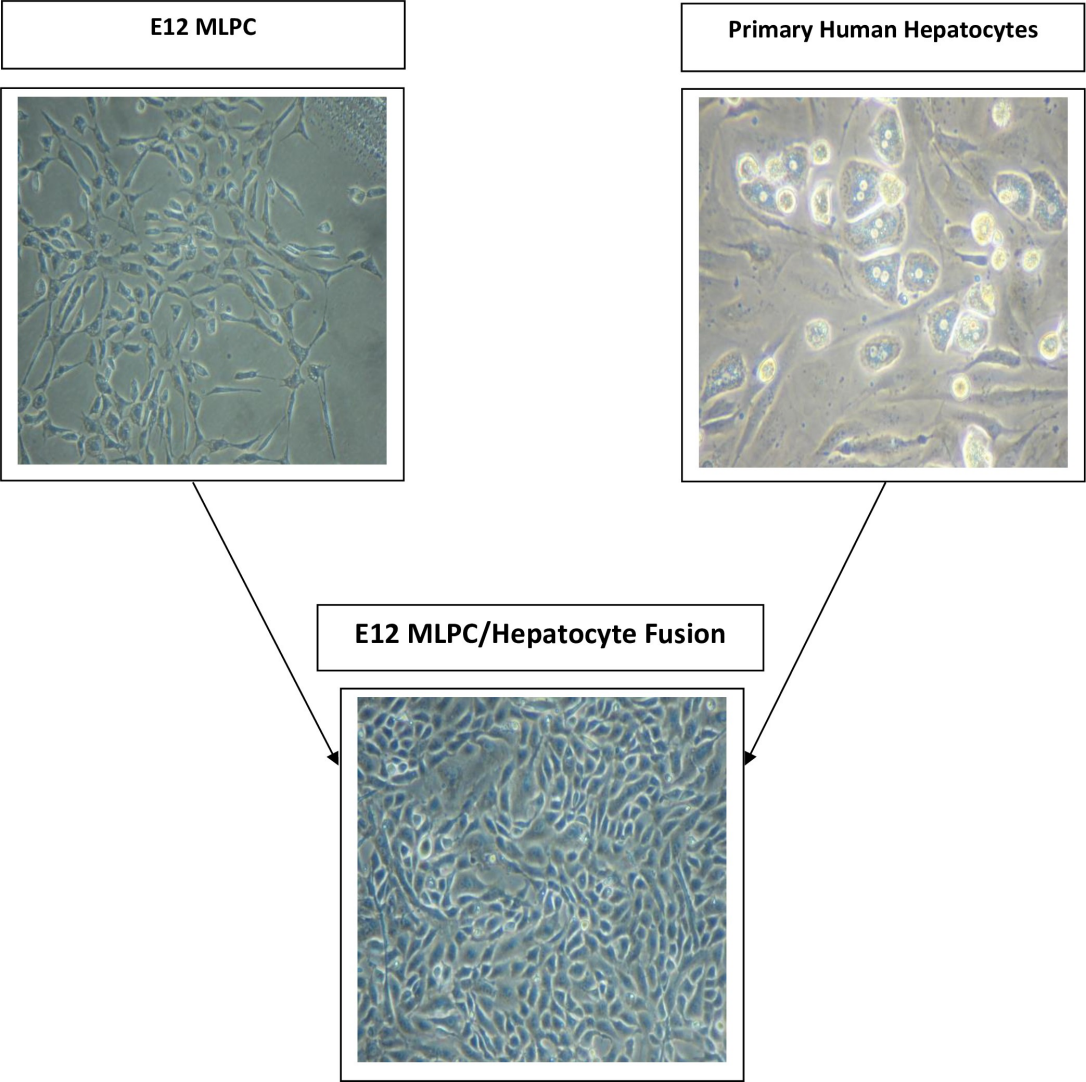
Phase contrast photomicrographs of E12 MLPC, primary hepatocytes and fusion cells of E12 MLPC.

The morphology of the various cells in this study is shown in phase contrast photos of the individual E12 cells, primary hepatocytes and the fused progeny of the two cell types (Fig 1). E12 MLPC cells are characterized by a typically spindle fibroblast-like morphology, single nucleus, obligate adhesion culture and a doubling time of ~ 24–27 hours. Primary hepatocytes are characterized by a roundish to square shaped morphology that appears like a cobblestone when cultured at high culture densities. PH are viable under recommended culture conditions for 5–7 days. Hepatocytes exhibited multiple nuclei in a significant proportion of plated cells. Fusion cells exhibited a cobblestone culture pattern with round, square and trapezoidal shaped cells. Individual cells were mainly mononuclear, or multi-nuclear with 2, 3 or 4 nuclei. A few individual cells expressed as many as 13 nuclei, but they did not appear to survive cryo-preservation and expansion. The multi-nuclear appearance of fusion cells was maintained through all freeze-thaw and expansion cycles. Fusion cells have been cultured for at least 12 months and have been shown to be bankable when cultured in the specific expansion medium described in ‘Methods and Materials’.

### TERT expression

**Fig 2:**
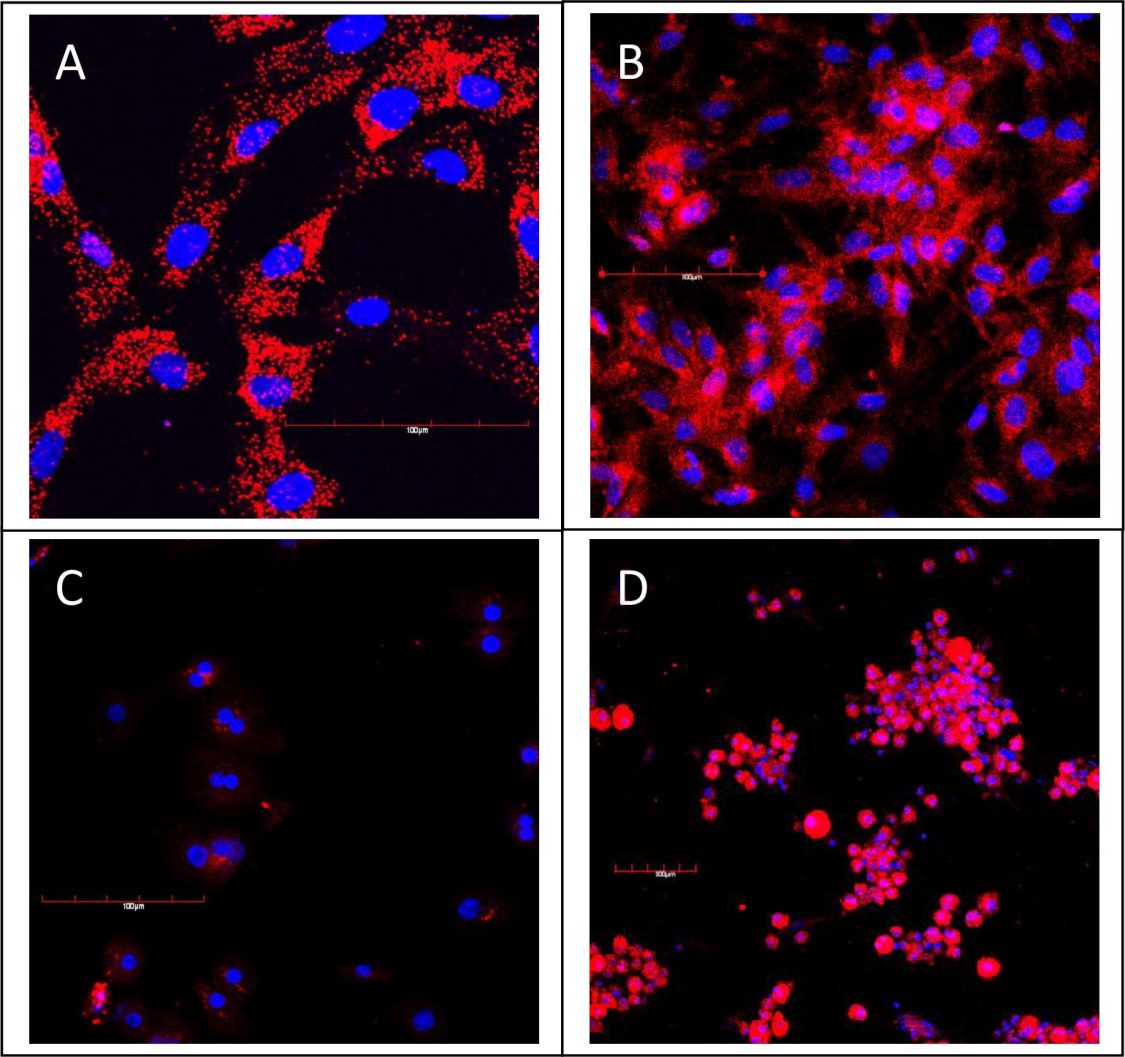
Confocal analysis of TERT Expression in MLPC E12 clone and human primary hepatocytes. (A) mRNA expression of TERT in MLPC E-12 clone analyzed by FISH analysis. (B) Detection of TERT expression by anti-TERT antibody in MLPC E12 clone; (C) normal human primary hepatocytes; and (D) MLPC/primary hepatocyte fusion cells. Each scale bar represents 100 μm.

The E12 cell line was analyzed for TERT expression by both FISH analysis and fluorescent immunohistochemistry. E12 cells expressed high levels of TERT specific mRNA in 100% of the E12 cells as shown by the presence of red markings in the cytoplasm of each cell (Fig 2A). The presence of the gene product of TERT by antibody analysis in E12 cells was demonstrated by positive red staining in the cytoplasm of each cell (Fig 2B). PH were shown to have low and sporadic expression of TERT by antibody analysis (Fig 2C). High expression of TERT was observed in 100% of the E12/PH fusion cells by antibody analysis as seen in Fig 2D.

### Confocal analysis of hepatocyte-specific markers

**Fig 3:**
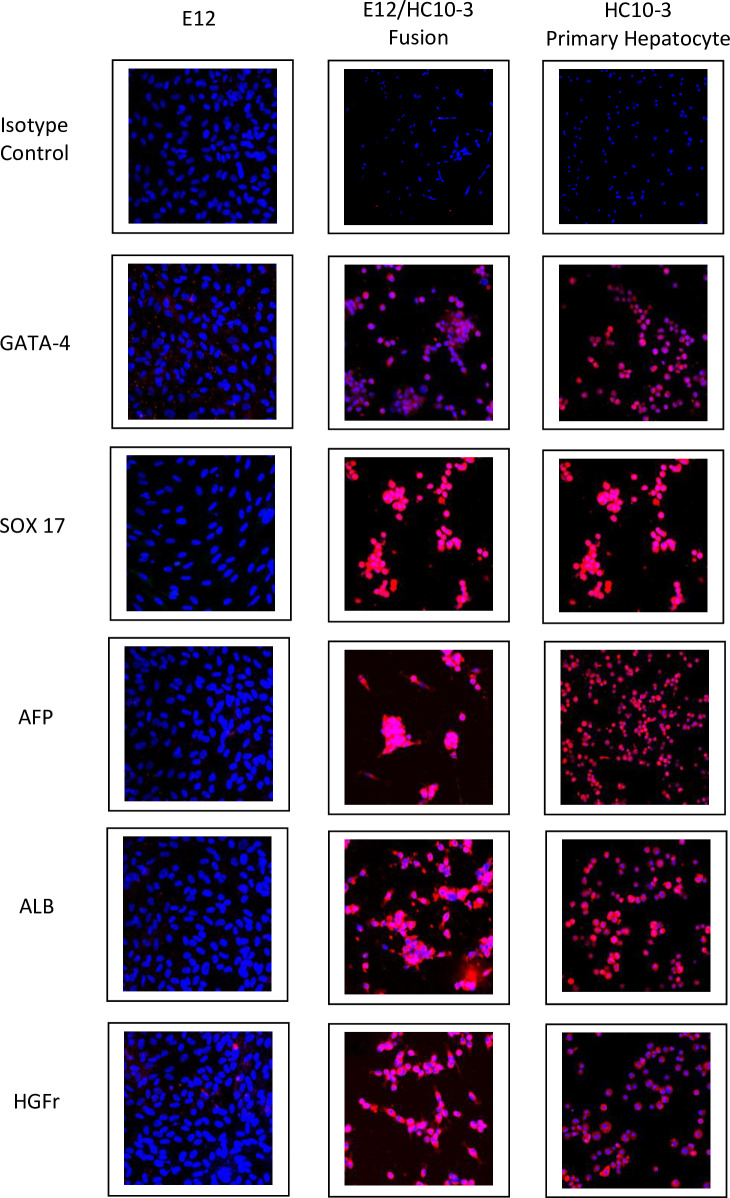
Expression of GATA4, SOX17, AFP, ALB and HGFr by the E12 MLPC clonal cell line (left column); E12/HC10-3 hybrid cell line (middle column); and HC10-3 primary hepatocytes (right column).

**Fig 4:**
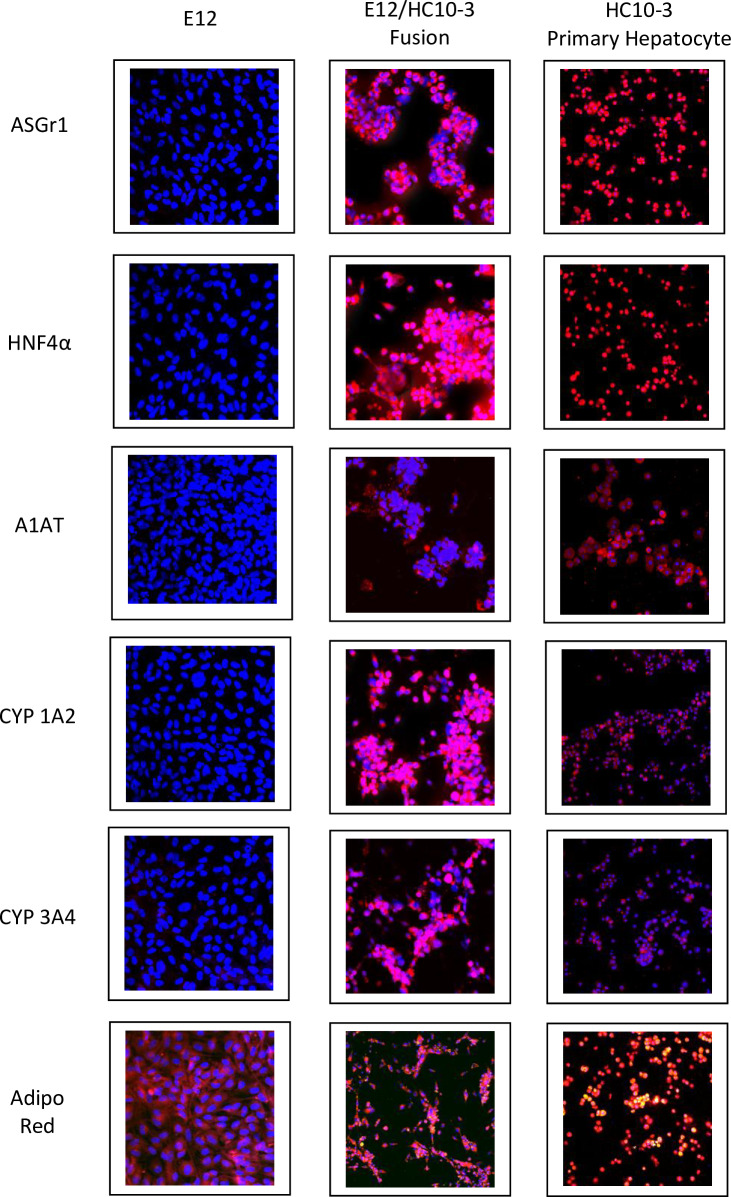
Expression of ASGr1, HNF4α, A1AT, CYP 1A2, CYP 3A4 and Adipo Red (triglycerides are seen as yellow dots) by the E12 MLPC clonal cell line (left column); E12/HC10-3 hybrid cell line (middle column); and HC10-3 primary hepatocytes (right column).

**Fig 5:**
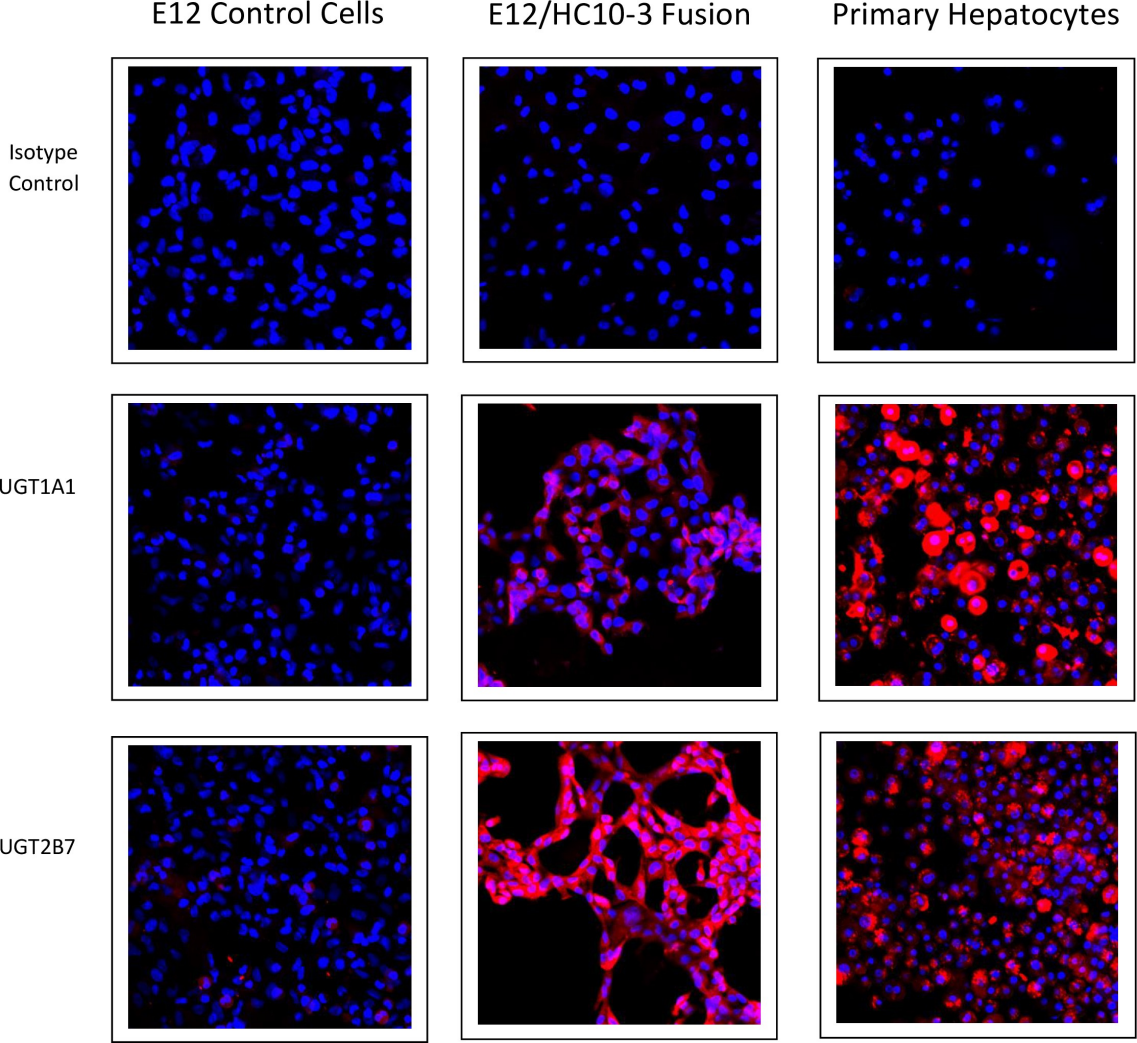
Expression of UGT1A1 and UGT2B7 in the non-fused E12 MLPC clonal cell line (left column), in the fused E12/HC10-3 hybrid cell line (middle column) and primary hepatocytes (right column).

Confocal analysis of E12 cells, PH and fusion cells is summarized in Table 1. Confocal images of the expression of hepatocyte markers are shown in Figs 3 and 4. Positive staining for the markers is demonstrated by detectable surface, cytoplasmic, or in the case of HNF4, nuclear staining, shown in red. Commitment to definitive endoderm is confirmed by positive staining for SOX17 and GATA-4. Further commitment to the hepatocyte phenotype was confirmed by other specific markers (Table 1). Those of note were the positive expression of albumin, nuclear HNF4, the ASGP receptor (Figs 3 and 4) and cytochrome P450 isotypes 1A2 and 3A4 indicative of mature hepatocytes (Fig 4). The expression of glucuronosyltransferase isoforms UGT1A1 and UGT2B7, critical enzymes in liver that are essential for the conjugation and subsequent elimination of potentially toxic xenobiotic and endogenous compounds, including bilirubin, was demonstrated by immunohistochemistry in both the PH anf the hybrid cells. In contrast, the native non-fused parental E12 cells showed no detectable expression (Fig 5).

**Table 1:** 

	TERT-MLPC E12	E12/Hepatocyte Fusion Cells	Primary Human Hepatocytes
Alkaline Phosphatase	Neg	Pos	Pos
Alpha fetoprotein	Neg	Pos	Pos
Albumin	Neg	Pos	Pos
C-reactive protein	Neg	Pos	Pos
Hepatocyte Growth Factor Receptor	Neg	Pos	Pos
Coagulation Factor VII	Neg	Pos	Pos
Coagulation Factor IX	Neg	Pos	Pos
Nestin	Neg	Pos	Pos
SOX 17	Neg	Pos	Pos
P450 CYP 3A4	Neg	Pos	Pos
P450 CYP 1A2	Neg	Pos	Pos
Asialo glycoprotein receptor 1	Neg	Pos	Pos
Hepatocyte Nuclear Factor 4	Neg	Pos	Pos
GATA-4	Neg	Pos	Pos
Alpha-1-antitrypsin	Neg	Pos	Pos
TERT	Pos	Pos	Weak in some cells
Adipo Red (triglycerides)	Neg	Pos	Pos
UGT1A1	Neg	Pos	Pos
UGT2B7	Neg	Pos	Pos

### Urea and albumin production

Production of urea and albumin are critical functions of hepatocytes. As summarized in Table 2, E12 MLPCs produced very low levels of both urea and albumin when compared to primary hepatocytes. Fusion cells produced both urea and albumin levels that were comparable to the levels expressed by the primary human hepatocytes.

**Table 2:** 

Analyte	E12 MLPC	Fusion Cells	Primary Hepatocytes
Urea (nmoles/ml)	5.72 ± 5.61	78.475 ± 24.11	28.72 ± 22.9
Albumin (pg/ml)	0.7745 ± 0.92	5706.59 ± 4845.52	15725.38 ± 5490.89

Values were standardized to 1 x 10^6^ cells

### PCR analysis

**Fig 6:**
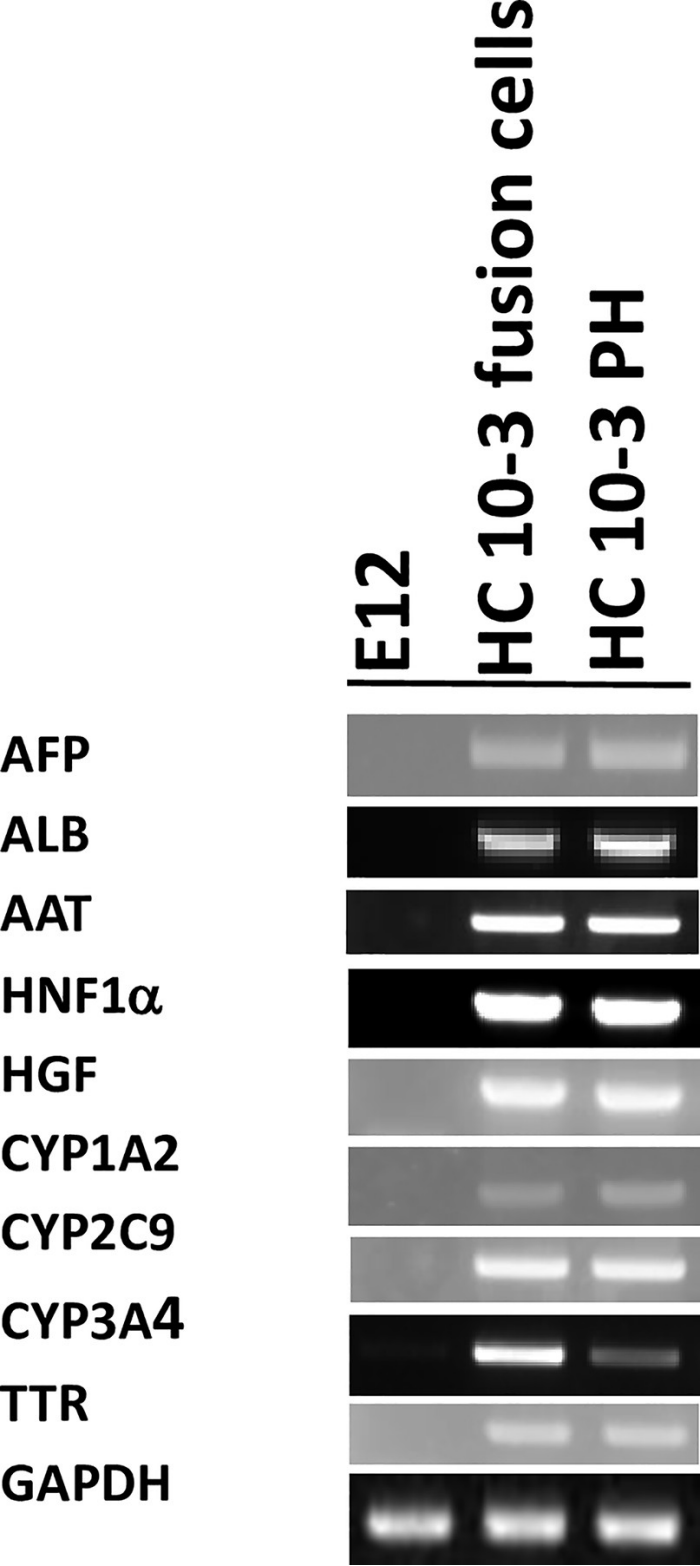
PCR analysis of the E12 MLPC clonal cell line; E12/HC10-3 hybrid cell line; and HC10-3 primary hepatocytes (PH) for liver-specific mRNA markers.

E12 MLPC, MLPC/hepatocyte fusion cells and primary hepatocytes were analyzed by PCR for liver-specific RNA isolates. RNA isolates were analyzed for the expression of alpha fetoprotein (AFP), α-1-antitrypsin (AAT), transthyretin (TTR), cytochrome P450 1A2 (CYP 1A2), cytochrome P450 3A4 (CYP 3A4), cytochrome P450 2C9 (CYP 2C9), hepatocyte nuclear factor 1 alpha (HNF1A), hepatocyte growth factor (HGF), albumin (ALB), and housekeeping gene glyceraldehyde-3-phosphate dehydrogenase (GAPDH). As shown in Fig 6, E12 MLPC exhibited none of the hepatocyte-specific markers, while both primary hepatocytes and MLPC/hepatocyte fusion cells similarly expressed each of the hepatocyte-specific markers.

#### Karyotype analysis

**Fig 7:**
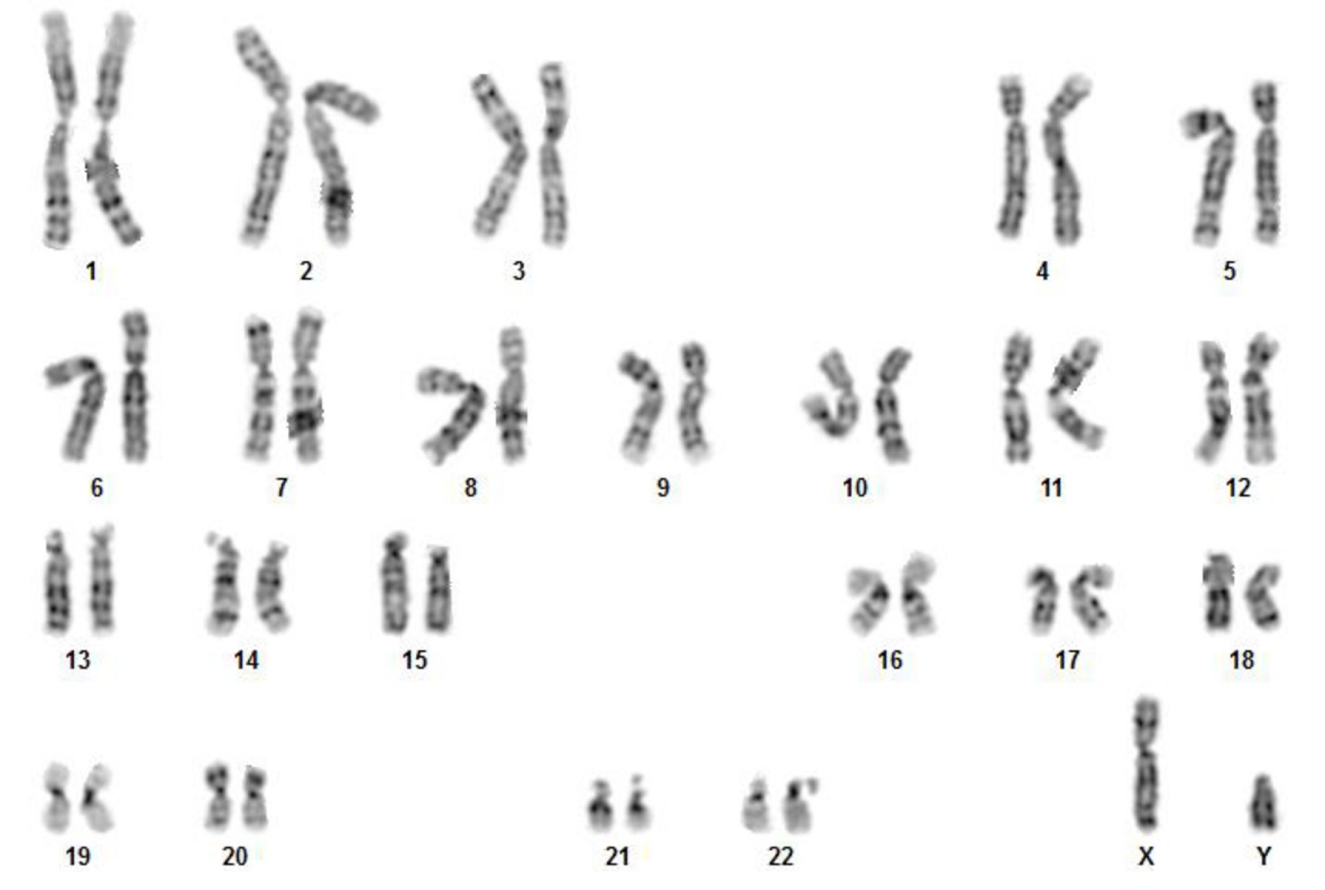
Karyotypic analysis of hepatocyte-like fusion cells.

Karyotypic analysis of the fusion cells showed no numerical or structural chromosomal abnormality associated with the over expression of TERT or the fusion process. The E12 MLPC cell line is from a male cord blood cell, the PH was from a female donor (Fig 7).

## Discussion

Livers and hepatocytes play crucial roles in the treatment of hepatic failure, and toxicology and drug metabolism studies, respectively. The need for both livers and hepatocytes is underserved by the lack of sufficient donors for both organs and cells. Simply stated, there is a critical shortage of donor livers suitable for transplantation. Additionally, livers found unsuitable for transplantation are used as the sources for isolated hepatocytes. This results in primary hepatocytes that have limited viability, decreasing functionality with age, inability to expand *in vitro*, significant variability on biological functions between different donor hepatocytes and finite supplies of quality hepatocytes [1,2]. Thus, other methods have been developed to generate cells with the functional characteristics of well-differentiated hepatocytes and the proliferative capacity to support large-scale production [19, 20]. Our results describe a novel cell type that exhibits hepatic functionality and proliferative capacity. The E12-TERT MLPC clonal cell line, possessing the best characteristics of both proliferative and differentiation capacity, were used throughout this study as the fusion partner for the primary hepatocytes.

MLPC are a subset of mesenchymal-like cells isolated from human umbilical cord blood. Experiments designed to develop clonal lines of MSC-like cells showed that only a small proportion of the cells were able to proliferate to quantities suitable for study. These clonal cells were capable of up to 80-population doublings and capable of differentiation to ecto-, endo- and mesodermal outcomes [3–6]. The remaining cell population was limited in their proliferative capacity (18–25 population doublings) and their ability to be differentiated to non-mesodermal outcomes. Over-expression of the TERT gene product by transfection resulted in a cell (E12 cell line) that has been cultured for over 12 years and maintains its capacity for differentiation to non-mesodermal outcomes. A study describing the *in vitro* differentiation of TERT-immortalized E12 cells into proliferative cells with hepatocytes functions is the subject of a separate report.

Previous publications have suggested that transfection with the TERT gene can functionally immortalize fetal liver cells or bone marrow-derived MSC without affecting their differentiation capacity. This was in contrast to more mature cell types that lost their proliferative capacity or their biological function with age [20–22]. It appears that inherent qualities present in fetal cells or stem cells enable them to achieve functional immortality via a single TERT gene. The E12 cell line exhibits those desirable qualities of proliferative and differentiation capacity with insertion of the TERT gene.

The results presented in this study demonstrate the advantage of fusion between the two cell types. E12 cells are negative for all hepatocyte-specific markers and positive for TERT. Hepatocytes are uniformly positive for hepatocyte-specific markers and mainly negative for TERT. The resultant fusion cells are uniformly positive for hepatocyte markers and TERT. This was further confirmed by PCR analysis where E12 MLPC were negative for all hepatocyte-specific RNA and the E12/HC10-3 fusion cells were uniformly positive for the hepatocytes-specific RNA as compared to human primary hepatocytes.

Functionality of resultant fusion cells was compared to the parental cell types, E12 MLPC and primary hepatocytes, by examining production of both urea and albumin. The E12 MLPC displayed very low levels of both urea and albumin production when compared to normal primary hepatocytes. In contrast, the E12/primary hepatocyte fusion cells produced significant levels of both urea and albumin, almost comparable for albumin, and in this limited study even higher levels of urea when compared to primary hepatocytes.

Bone marrow and cord blood cells have been suggested as potential sources of cells for liver regeneration based on several observations. Specifically, donor cells from a bone marrow or cord blood transplant were found functioning as hepatocytes in the liver of recipients with the opposite sex type of the donors [23, 25–28]. Another study described a similar phenomenon in the pancreas of cord blood allograft recipients [24]. A number of studies were designed to determine whether the transformation of the donor stem cells was due to transdifferentiation of the stem cells to a hepatocyte phenotype, or due to fusion of the donor cells with the recipient’s liver cells [19,23]. Sato, et al. transplanted human mesenchymal stem cells directly into rat livers [25] and reported that the resultant albumin-producing donor derived cells in the liver were not the result of cell fusion, but rather due to transdifferentiation of the MSC to hepatocytes. In that same study, the successful implantation rate was less than 1% of the injected cells (possibly reflecting the rarity of these cells within the MSC population). Nevertheless, the results suggested that trandifferentiation was a legitimate mechanism for appearance of donor-originated hepatocytes in the recipient. While some studies have suggested transdifferentiation to hepatocytes [23, 26–28], others have implicated cell fusion as the source of donor cells in the liver [29–34]. If the E12 TERT MLPC, described herein, are reflective of a small population of stem cells with characteristics of MSC-like cells within bone marrow, then both transdifferentiation and fusion are possible and not necessarily exclusive to the occurrence of functional donor cells in the liver of recipients.

Our results provide a basis for the development of immortalized liver cell lines with large-scale production capabilities for general drug metabolism and toxicology studies. In addition, it is conceivable that this methodology could be employed to create immortalized hepatocyte-like cell lines with specific pathologies to provide a renewable source of cells for research and development of new therapies. Immortalized and potentially infinitely expandable populations of clonally-derived disease-specific hepatocytes could provide an important tool for the development of new therapies for those diseases.

The successful fusion of primary hepatocytes with an immortalized cord blood-derived stem cell line (E12) resulting in a stable proliferative cell with the characteristics of hepatocytes might provide a procedural pathway for the development of other organ/cell-specific immortalized cell lines. Studies describing the *in vitro* differentiation of various stem cells to functional organ-specific cells (like hepatocytes) are characterized by large variances in the time to differentiation, growth factor compositions, viability and more. *In vitro* differentiation protocols suffer from the current inability to mimic all of the tissue-specific and time-dependent differentiation signals that occur during the development of the fetus. The ability to by-pass those complicated and time-consuming experiments necessary to clearly define the proper sequences of growth factors, culture conditions and the contributions of extra-cellular matrices could speed the development of organ and cell-specific immortalized cell lines. The application of a four decades-old simple and highly repeatable methodology that has been successfully applied to generate monoclonal antibodies could shortcut the development time for differentiated cells, and tissues. Employment of this methodology could enable similar cell fusions with other cell types creating immortalized cell lines with the characteristics of pancreas, neurons, and numerous other cell types.

Finally, a renewable source of functional hepatocyte-like cells could contribute significantly to the development of artificial liver support systems. One could imagine an extracorporeal system containing cells that would enable patients with liver failure to regenerate their own livers or survive as a bridge to transplant [34,35].

## Conclusion

We report observations with regards to TERT-immortalized MLPC. They were capable of conferring immortality upon fusion with human primary hepatocytes. The resultant fusion cells retained the biological activity of the primary hepatocytes and the immortality of the TERT-immortalized MLPC. Fusion cells retained 100% expression of hTERT as well as expression of mRNA, urea and albumin production, and intracellular marker expression characteristic of primary hepatocytes. These cells have been serially cultured for an extensive period without loss of hepatocyte biological activity nor their immortality.

## Supporting information

S1 Fig (DOCX)

S1 Table Primer sequences for PCR. (DOCX)
