# Supplementary material for: Development of immortalized human hepatocyte-like hybrid cells by fusion of multi-lineage progenitor cells with primary hepatocytes
Source: PLoS One. 2020 Jun 4;15(6):e0234002. doi: 10.1371/journal.pone.0234002 (PMC7272032; doi:10.1371/journal.pone.0234002)
Supplement: S1 Fig — (DOCX) [file pone.0234002.s001.docx]

**Figure 6 Supplementary data**

Collins DP, Hapke JH, Aravalli RN, and Steer CJ. “Development of immortalized human hepatocyte-like hybrid cells by fusion of multi-lineage progenitor cells with primary hepatocytes“.

Raw images of RT-PCR blots from Figure 6 are shown below. PCR products of each hepatocyte-specific gene as well as that of the housekeeping gene GAPDH were resolved by agarose gel electrophoresis as described in the ‘Materials and Methods’ section. Lane 1: E12 cell line; Lane 2: HC 10-3 fusion cells; and Lane 3: HC 10-3 primary hepatocytes.


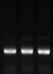


**GAPDH**


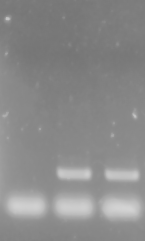


**TTR**


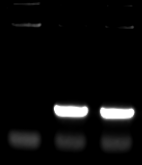


**HNF1α**

Primer dimer

1 2 3

1 2 3

1 2 3


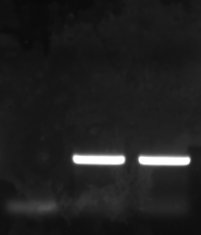


**AAT**

1 2 3


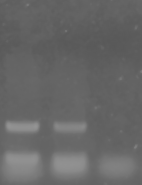


**CYP1A2**

1 2 3

Primer dimer

Supplementary data continued…

Collins DP, Hapke JH, Aravalli RN, and Steer CJ. “Development of immortalized human hepatocyte-like hybrid cells by fusion of multi-lineage progenitor cells with primary hepatocytes“.


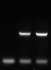

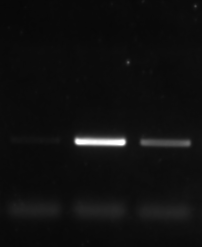

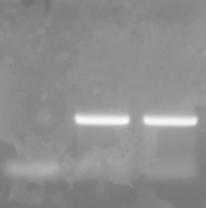

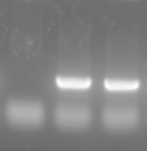


**HGF**

**ALB**

**CYP3A4**

**CYP2C9**

**AFP**


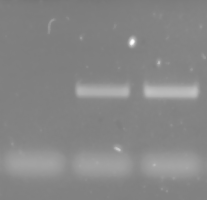


Primer dimer

Primer dimer

1 2 3

1 2 3

1 2 3

1 2 3

1 2 3
