# Supplementary material for: Development of immortalized human hepatocyte-like hybrid cells by fusion of multi-lineage progenitor cells with primary hepatocytes
Source: PLoS One. 2020 Jun 4;15(6):e0234002. doi: 10.1371/journal.pone.0234002 (PMC7272032; doi:10.1371/journal.pone.0234002)
Supplement: S1 Table — (DOCX) [file pone.0234002.s002.docx]

**Supplemental Table 1**

Primer sequence Reference

AFP         F: 5′-TGCAGCCAAAGTGAAGAGGGAAGA-3′   13

                  R: 5′-CATAGCGAGCAGCCCAAAGAAGAA-3′

AAT           F: 5'-ACTGTCAACTTCGGGGACAC-3' 14

                    R: 5'-CATGCCTAAACGCTTCATCA-3'

TTR           F: 5'-TCATCGTCTGCTCCTCCTCT-3' 14

                     R: 5'-AGGTGTCATCAGCAGCCTTT-3'

CYP1A2     F: 5'-CAATCAGGTGGTGGTGTCAG-3' 15

                     R: 5'-GCTCCTGGACTGTTTTCTGC-3'

CYP3A4     F: 5'-AAGTCGCCTCGAAGATACACA-3' 14

                     R: 5'-AAGGAGAGAACACTGCTCGTG-3'

CYP2C9      F: 5'-GGACAGAGACGACAAGCACA-3' 15

                     R: 5'-CATCTGTGTAGGGCATGTGG-3'

HNF1A     F: 5'-TACACCACTCTGGCAGCCACACT-3' 16

                    R: 5'-CGGTGGGTACATTGGTGACAGAAC-3'

GAPDH   F: 5′-GCACCGTCAAGGCTGAGAAC-3′ 17

                  R: 5′-ATGGTGGTGAAGACGCCAGT-3′

HGF        F: 5′-GTAAATGGGATTCCAACACGAACAA-3′ 17

                  R: 5′-TGTCGTGCAGTAAGAACCCAACTC-3′

ALB         F: 5'-CTTATTCCAGGGGTGTGTTTCG-3' 18

                  R: 5'-CGATGAGCAACCTCACTCTTGTG-3'

Primer sequences for PCR analysis
